# Supplementary material for: Development of an HPLC-MS/MS Method for the Determination of Alkaloids in Lupins
Source: Molecules. 2023 Feb 5;28(4):1531. doi: 10.3390/molecules28041531 (PMC9967435; doi:10.3390/molecules28041531)
Supplement: Supplementary file 1 [file molecules-28-01531-s001.zip › molecules-2163438-supplementary.pdf]

Supplementary material

Development of HPLC-MS/MS method for the determination of alkaloids profile in lupins

Fabiola Eugelio [1], Sara Palmieri [1], Federico Fanti[1,*], Luana Messuri[1], Alessia Pepe[1], Dario Compagnone [1], and Manuel Sergi [2]

[1]Department of Bioscience and Technology for Food, Agriculture and Environment, University of Teramo, Via Renato Balzarini 1, 64100 Teramo, Italy

[2]Department of Chemistry, Sapienza University of Rome, Piazzale Aldo Moro 5, 00185 Rome, Italy

[*]Correspondence: ffanti@unite.it

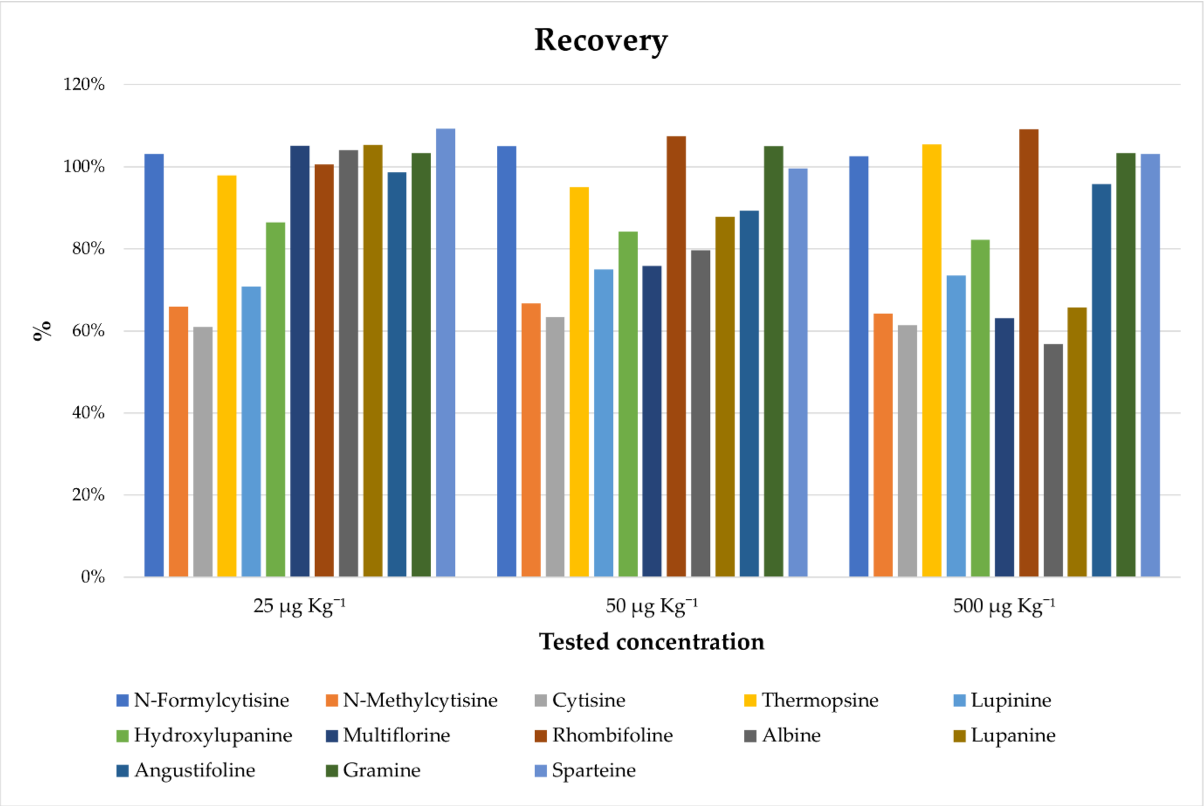

Figure S1. Results of recovery evaluation obtained from validation tests are shown.

Table S1. LAs identified and quantified in *L. albus* L. raw seeds, characterized by different size and different agronomical treatments. Data are reported in mg Kg^{-1}.

Analytes	Raw seeds 11/13 mm (mg Kg^{-1})	Raw seeds 13/15 mm (mg Kg^{-1})	Raw seeds 15/17 mm (mg Kg^{-1})	Raw seeds 15/17 mm Bio (mg Kg^{-1})
Lupanine	25400.85±201.81	19685.45±967.61	18007.35±946.53	31025.80±1703.07
Multiflorine	1276.42±367.12	1524.96±96.34	1176.75±62.15	2367.27±388.33
Albine	691.8±55.34	502.08±52.22	386.73±31.64	818.50±30.41
Hydroxylupanine	594.43±562.96	220.25±67.67	126.10±31.40	443.18±0.81
Angustifoline	288.93±40.57	192.15±3.55	190.52±13.15	306.98±13.39
Sparteine	164.15±30.41	60.96±18.17	71.03±17.15	68.98±29.02
Total content	28416.56±1258.22	22185.83±1205.55	19958.47±1102.02	35030.70±2165.04

Table S2. Chemical properties and supplier information of the target standard alkaloids.

	N-Formylcytisine	Cytisine	(+)-Hydroxylupanine	N-Methylcytisine	(−)-Lupinine	Albine	(−)-Angustifoline
	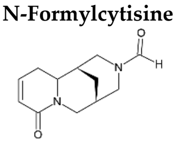	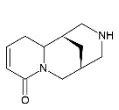	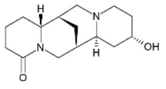	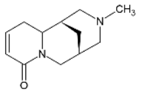	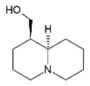	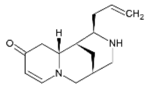	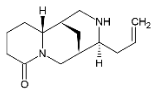
Nominal mass (Da)	218.25	190.24	264.36	204.27	169.26	232.32	234.34
Molecular formula	$C_{12}H_{14}N_2O_2$	$C_{11}H_{14}N_2O$	$C_{15}H_{24}N_2O_2$	$C_{12}H_{16}N_2O$	$C_{10}H_{19}NO$	$C_{14}H_{20}N_2O$	$C_{14}H_{22}N_2O$
CAS	53007-06-0	485-35-8	15358-48-2	486-86-2	486-70-4	53915-26-7	550-43-6
logP	0.1	0.2	0.6	0.7	1.2	1.3	1.4
Supplier	LGC Standards Srl	Merck Life Science Srl	Vinci-Biochem Srl	LGC Standards Srl	Merck Life Science Srl	Phytoplan	Phytoplan
Purity	98%	≥99%	≥98%	97%	≥98%	>95%	>95%

	Multiflorine	Thermopsine	(+)-Lupanine	Rhombifoline	Gramine	(+)-Sparteine
	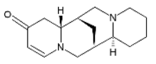	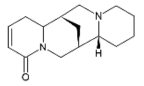	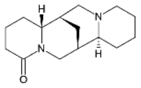	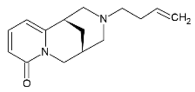	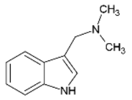	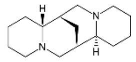
Nominal mass (Da)	246.35	244.33	248.36	244.33	174.24	234.38
Molecular formula	$C_{15}H_{22}N_2O$	$C_{15}H_{20}N_2O$	$C_{15}H_{24}N_2O$	$C_{15}H_{20}N_2O$	$C_{11}H_{14}N_2$	$C_{15}H_{26}N_2$
CAS	529-80-6	486-89-5	550-90-3	529-78-2	87-52-5	492-08-0
logP	1.5	1.6	1.6	1.8	1.8	2.5
Supplier	Phytoplan	Vinci-Biochem Srl	Vinci-Biochem Srl	Clinisciences	Merck Life Science Srl	Merck Life Science Srl
Purity	>95%	≥98%	≥95%	≥98%	≥99%	≥98%

Table S3. MS/MS parameters of the selected analytes for the MRM acquisition; m/z value of precursor compounds in first quadrupole (Q_1) is reported as $[M+H]^+$; also, m/z values of the ion fragments in the third quadrupole (Q_3) are reported.

Analytes	R_t (min)	Q_1 (Da)	DP (V)	EP (V)	Q_3 (Da)	CE (V)	CXP (V)
N-Formylcytisine	2.38	218.9	110	9	148.0	33	11
					160.1	34	10
N-Methylcytisine	2.88	204.9	90	7	146.1	29	5
					107.9	28	7
Cytisine	2.90	190.8	95	10	148.1	28	5
					133.0	45	10
Thermopsine	3.17	245.1	98	11	98.1	38	10
					70.1	66	7
(-)-Lupinine	3.23	170.0	95	11	152.0	32	10
					124.0	38	10
(+)-Hydroxylupanine	3.25	265.0	122	10	247.2	36	10
					148.1	50	10
Multiflorine	3.34	247.1	16	10	112.2	35	4
					70.1	56	5
Rhombifoline	3.50	245.1	90	10	98.2	31	8
					203.1	25	8
Albine	3.51	233.0	24	11	112.1	29	6
					138.0	25	9
(+)-Lupanine	3.52	248.9	45	11	136.1	41	4
					114.0	38	8
Angustifoline	3.73	235.1	46	12	193.0	27	6
					112.1	37	6
Gramine	4.22	175.1	38	10	130.0	18	4
					46.1	16	3
(+)-Sparteine	4.26	235.0	45	11	98.1	49	8
					233.1	38	8
